# How to study political parties: from civil society to the state and back. The Peter Mair lecture 2023

**DOI:** 10.1080/07907184.2024.2323486

**Published:** 2024-05-09

**Authors:** Nicole Bolleyer

**Affiliations:** Geschwister Scholl Institute of Political Science, Ludwig-Maximilians-Universitat, Munich, Germany

**Keywords:** Party organization, cartel party theory, civil society, party-state relations, membership organizations

## Abstract

Cartel party theory has put the study of party-state relations high on the research agenda, deliberately shifting the focus of researchers away from an understanding of political parties on the basis of their relationship with civil society or, indeed, as part of civil society itself. As fruitful as this reorientation has been, this essay argues that the resulting emphasis on ‘parties as governors’ has produced downsides of its own. Cartel party theory reinforced a separation of the study of parties from the study of other membership organizations considered the very fabric of civil societies. While their governing role makes parties distinct from the latter, I argue that what parties do and how they function should be also assessed against other organizations such as interest groups or associations through which citizens engage in joint activities including but not restricted to political interest representation. The contention is that bringing the study of party back to civil society will generate a broader understanding of the possible roles political parties can (or cannot) play in contemporary democracy than an exclusive study of party as a separate genus.

This essay on Peter Mair’s contribution to research on political parties as subfield of comparative political science starts out from observations articulated by various colleagues since Peter’s untimely death. Though Peter’s work was and is highly influential, to this day fundamentally impacting the comparative study of political parties, it did not shape research through what is by now the dominant mode of conducting political science – by generating clear-cut hypotheses that straightforwardly lend themselves to ‘empirical “testing”’. As put by Nick Aylott (2021) when reviewing Peter’s and Richard S. Katz’ 2018 OUP book that was published posthumously: ‘Perhaps we need to accept that some things in politics (…) are just hard to get at empirically. That, of course, makes the cartelization thesis stimulating and frustrating in equal measure’ (Aylott, 2021, p. 598). Similarly, when specifying the various empirical tendencies associated with party organizations’ developments into ‘cartel parties’ in a volume published in Peter’s honor (e.g. public funding of parties, their withdrawal from society), Stefano Bartolini and Hans Daalder (2014) point out the following: ‘[i]t is not easy to single out the most important element triggering these developments and the causal constellation should be primarily seen as a “syndrome”’ (p. 33). They further stress, however, that in his ‘writing about the “cartel party” the word “state” is the word that most prominently and often appears. Unquestionably, the core of the transformation lies in the new relationship between the once “private” association party and the branches, finance, and institutions of the state.’ (Bartolini & Daalder, 2014, p. 33).

I am going to argue that ‘the state’ as the core of this empirical transformation many mainstream parties have (at least in some respects) undergone in modern democracy, lies also at the heart of an intellectual shift that has fundamentally shaped how we tend to conduct party research to start with. This shift one might call the ‘statist turn’ of party research replacing the well into the 1990s more dominant society-centered perspectives on parties as a subject of study. One expression of this ‘turn’ is that while to this day controversies are on-going over the extent to which specific hypotheses derived from Peter’s work on party system cartelization and the cartel party model hold in specific country settings (see for a recent study on Sweden, Enroth & Hagevi, 2018), it is little questioned that a transformation – parties (one way or the other) having moved away from society and towards the state – has taken place. Emulating Bartolini and Daalder’s (2014) use of metaphors (speaking about the drivers underpinning cartel party tendencies as part of a ‘syndrome’), one might say that while debates over the exact nature of how particular symptoms manifest themselves in different patients is on-going, the very presence of the underlying illness as such is little contested.

Peter’s arguments about the cartel party as a new form of party organization were not just influential in a substantive sense but on a fundamental level shifted how parties as a subject of comparative study since the 1990s were thought about in scholarly work. Offering a state – rather than society-centered perspective on parties, he altered the dominant approach to how the phenomenon ‘political parties’ ought to be thought about and studied. Peter’s work thereby has left its mark on party research much more pervasively than is visible in works that explicitly refer to him or strands of research that deals with one of the themes closely associated with the cartel party as a model, of which there were many (e.g. the dependence of parties on state resources, state regulation of parties, the changing distribution of power within political parties benefitting the party in public office at the cost of the membership organization, the formal empowerment of rank-and-file members which he expected to lead a displacement of activists) (e.g. Katz & Mair, 1993, 1994, 1995, 2009; Mair, 1994; Bartolini & Daalder, 2014; van Biezen, 2014; Bardi, 2022).

In the following, I will specify this ‘statist turn’ of party research, illustrate its roots in Peter’s earlier work and how it has, in my view, shaped the nature of mainstream party research until today. Often parties’ close proximity to the state seems to implicitly function as a defining feature of what contemporary mainstream parties *have become*, thereby separating their study from that of other societal organizations mobilizing collective interests. Such a division of labor between party, group, and movement scholars is often fruitful but sometimes prevents us from taking a broader, society-centered perspective that might allow us to explore the implications of societal change more suitably than a party-centered approach. I will then talk about how different strands of research have started to challenge this tendency and will conclude with an illustration of how treating parties as one type of membership organization alongside others might reveal their special status in the societal realm more effectively than taking their distinctiveness as a given.

## From civil society to the state – the intended legacy

The ‘statist turn’ of party research has substantively and analytically relocated political parties as a subject of study away from civil society into the realm of state institutions, with fundamental conceptual, theoretical and empirical consequences for party research. It goes back to a plea that can be repeatedly found in Peter’s pieces from the early 1990s onwards, some single-authored, some co-authored with Richard S. Katz. This plea stresses the need to change the dominant perspective on political parties, a perspective he claimed to be outdated and unhelpful for understanding the latter’s role in modern democracy. To quote Peter’s and Dick’s best known article published in *Party Politics* in the mid-1990s: ‘[t]he view that parties are to be classified and understood on their basis of their relationship with civil society’ is ‘ill founded’ and has led ‘to undervalue the extent to which differences between parties may also be understood by reference to their relations with the state.’ (1995, p. 293). This line of argument was tied to a concern that researchers had insufficiently recognized the distinctiveness of parties, ‘the obliteration of much of what was distinctive about party as a mode of interest representation, and thus also the obliteration of the distinction between party, on the one hand, and other forms of interest representation, on the other’, as highlighted by Peter in his 1992 Stein Rokkan lecture (1993, p. 131). Ignoring their distinctive nature was bound to become more problematic over time, as parties’ growing proximity to state institutions reinforced their special status and separation from the societal realm. As Peter stated later on in his prominent *Ruling the Void:* ‘Real opposition’ is exercised by political movements, while “parties are either governing or waiting to govern. They are now all in office”’ (2013, pp. 88–89).

In essence, in modern democracy parties are no longer part of civil society and therefore should not be approached, conceptualized or theorized as such. As Peter repeatedly took inspiration from Giovanni Sartori, he was acutely aware that how we conceptualize the political phenomena we study has far-reaching consequences. Thus, how we (implicitly or explicitly) address the ‘what is’ question when studying parties was bound to be an important concern to him (Mair, 2008, p. 72).

It is fair to say that by now the ‘ill founded view that parties are to be classified and understood on their basis of their relationship with civil society’ (Katz & Mair, 1995, p. 293) has been overcome. And there is no need to reiterate here the numerous fruitful avenues of innovative research that were inspired by the ‘statist turn’ shifting our attention to party-state relations. The benefits for especially research on party organization as a subfield in comparative politics were vast (Bartolini & Daalder, 2014; van Biezen, 2014). Yet the overcoming of the tacit (and too little reflected upon) assumption that parties are best understood as private associations has by now led to its own tacit assumptions, effectively reversing the situation Peter problematized in the 1990s.

Nowadays, it is no longer society-centered perspectives on parties that ‘run through the vast variety of typologies and analyses (both normative and empirical) that have been presented’ (Katz & Mair, 1995, p. 5). The statist perspective has become our new fall-back assumption, with broader society-centered perspectives on parties requiring justification instead. The dominant baseline is that parties – given ‘an ever closer symbiosis between parties and the state’ (Katz & Mair, 1995, p. 293) and as they ‘are either governing or waiting to govern’ (Mair, 2013, pp. 88–89) – are fundamentally distinct from other parts of organized civil society. They are no longer just a ‘primus inter pares’ in the societal realm as Rosenblum argued starting out from an overarching frame that linked the study of parties to those of other parts of organized civil society (2000a, 2000b). They are unique. And if parties are unique and no longer part of society, how can we usefully compare them to interest groups or civic associations?

What seems to have happened on a conceptual level is that treating parties’ location within state institutions as a (rarely questioned) starting assumption (instead of a *potential* state) invites a conflation of two closely related claims that predetermine our answer to the ‘“what is” question’ when embarking on new research projects and specifying parties as our ‘basic unit of thinking’ (Mair, 2008, p. 72). The first claim relates to what parties have become in modern democracy (e.g. according to cartel party theory a part of the state apparatus). The second claim relates to how parties as a class of organization should be conceptualized in light of particular aspects related to their functioning or activities that might interest us when developing new research. Keeping the two types of claims distinct is important as even if hypotheses derived from the ‘cartel party model of party organization’ were uniformly confirmed with regard to mainstream parliamentary parties across consolidated democracies, let’s say, the analytical lens to generate such research is not necessarily suitable to study parties as organizations *per se*.

Somewhat downplayed in light of its prominence, cartel party theory was no theory on political parties in general and did not try to be, becoming clear especially in Peter’s later work on populist parties (2002). From the start, it had been tailored to major parties with parliamentary representation (as was Kirchheimer’s catch-all model as one of its ‘predecessors’), not only visible in Peter’s work but was shared by those criticizing it. Ruud Koole argued in his critique to the seminal 1995 *Party Politics* article outlining the cartel party model of party organization that, irrespective of any closer relations to the state, parties ‘continue to possess a quasi-monopoly on the recruitment of political personnel’ (1996, p. 514). It is such monopoly that makes them distinct from other parts of organized civil society such as interest groups, non-profits or associations and rationalizes a close connection with state institutions. And this distinctiveness becomes the more consequential for party research overall, the more we equate being a party with having the foot in the door towards governing and *actually* placing political personnel within state institutions. Yet, taking the latter as what parties have become in modern democracy and answering the ‘“what is” question’ accordingly, we have effectively narrowed down our analytical scope to those parties with coalition potential (Sartori, 1976), while still claiming to theorize and study parties *as such*. Holding the monopoly in the recruitment of political personnel (rather than just nominating electoral candidates of whom only few will end up as political personnel) of course not only requires being a party by running elections (Sartori’s much used definition of parties). It presupposes (at the least) that parties do win parliamentary seats – overcoming the threshold of representation – which most organizations that qualify as parties in Sartori’s sense rarely achieve (Pedersen, 1982) and some organizations running elections might not even aspire to (Farrer, 2017). The latter is clearly in tension with a notion of parties that ‘are either governing or waiting to govern’ (Mair, 2013, pp. 88–89).^1^

To be clear, there is nothing wrong with formulating a theory of party organization that is tailored to those parties operating in state institutions that most scholars consider as most relevant to democracy and it’s functioning anyhow.^2^ As Kirchheimer’s catch-all party (1965) or Panebianco’s electoral-professional party (1988), parties that increasingly resemble cartel parties tended to operate in state institutions all along. That to the latter the state has become even more important, while stable ties to society have been declining, has far-fetching consequences for democratic governance. Yet conceptually speaking it is useful to keep in mind that ‘forming part of the state apparatus’ requires the *actual* not just the *aspired* recruitment of political personnel into public institutions and that the monopoly held ‘by parties’ over such recruitment is held be a minority of those organizations that qualify as party organizations to start with.

## The unintended legacy of parties as separate and unique subject of study

Whether or not we define the class of political parties with reference to a property that party organizations generally possess such as the classical feature of nominating electoral candidates (Sartori, 1976), a state-centered approach to parties might still be a suitable focus for most research questions party scholars might decide to examine. But – echoing Peter’s repeated critique of the field’s ill-founded focus on party-society relations – it should not function as a default option. Today’s tacit assumption in party research seems to be that approaching political parties as part of civil society ignores the fundamental distinctiveness of what (most) parties have become in advanced democracies or, even worse, ignores what they have anyway never been in new democracies. Taken to the extreme, this might mean that a society-centered perspective is fundamentally unsuitable to motivate research on political parties. If such unsuitability was rooted in a general misunderstanding of or ignorance towards the very nature of political parties in modern democracy, it of course would apply *per se*, i.e. irrespective of the research question we might start out from.

This is not to say that tendencies towards ‘separating out’ party research from organization research broadly defined were not also linked to wide-spread pressures towards specialization affecting the social sciences overall, pressures especially on junior scholars towards finding one’s own niche with a clearly defined target audience. Research ignoring established boundaries between subfields easily falls in between stools and tends to be more difficult to publish, not the least because those boundaries are often there for good substantive reasons. But it is important for the production of knowledge that those boundaries remain permeable and we, once in a while, reflect upon them and their repercussions, as Peter did back in the 1990s with fruitful consequences for party scholarship.

Trying to do this, it materializes that part of the ‘statist turn’ of mainstream party research – ‘mainstream’ meaning both ‘dominant’ and ‘focused on the study of mainstream parties' – is a shift away of the field from broader comparisons able to encompass different organizational types such as interest groups, churches or civic associations. Such broader perspectives require a treatment of parties as comparable (and in some fundamental sense similar) to other societal organizations and requires us to distance ourselves from the idea that parties – given their special role in the state – are ‘too distinctive’ for their functioning and behavior to be theorized and examined alongside the latter. Encompassing society-centered perspectives on parties are at odds with state-centrism as the substantive core of the cartel party model (Bartolini & Daalder, 2014, p. 33), which in turn – by reinforcing claims of distinctiveness – invited a party-centrism in both a theoretical and analytical sense, two tendencies which are not inevitably linked but clearly complement each other.

An unintended reinforcement followed which was related to how the critique of an ‘ill-founded focus on party-society relations’ was addressed, namely by presenting the cartel party model as embodiment of an alternative, more fruitful perspective. Peter’s critique of ‘society-centrism’ was closely tied to a critique of the mass party model, a type of party organization with firm societal roots. This by now (supposedly) outdated model of party organization was reliant on a vision of social structure absent in post-industrial societies, thereby providing an unsuitable benchmark for what parties realistically can or ought to be in modern democracy (Katz & Mair, 1995, p. 293). However convincing the this argument was, the cartel party model as a heuristic device was thereby placed in the context of an on-going debate around which party models succeeded *each other* over time as societies transformed towards greater individualization, fragmentation and weakening collective identities. One might argue that bringing the critique of the mass party model as misplaced ‘gold standard’ (rooted in societies that no longer existed) to its logical conclusion would have required to move away from a longitudinal perspective altogether that invited a discussion of the cartel party model *in contrast to other party models*, be it Michels’ mass party (1915), Kirchheimer’s catch all party (1965) or Panebianco’s electoral-professional party (1988). Doing so implicitly tied state centrism to party centrism, keeping consecutive party research focused on how contemporary (mainstream) parties have changed as compared to what they once were or, more recently, how they differ from challenger parties (Meguid, 2008; De Vries & Hobolt, 2020).

A departure from a longitudinal perspective could have re-orientated the debate more towards whether and, if so, how party organizations operating in today’s increasingly individualized, fragmented or polarized societies try to assure their survival, establish ties to citizens or engage in participatory or representative activities *as compared to membership organizations* in principle able to constitute linkages that face similar challenges. To genuinely appreciate political parties’ role(s) in contemporary democracy, whatever it is parties do or do not do in the societal realm and whether they do it less or less well than when society was different, seems to be less crucial than whether or not there are other organizations or participatory channels that do it better or whether, alternatively, whatever was discussed as party decline or failure was only one expression of a broader societal transformation of organized civil society at large. How parties fare in contrast to alternative channels has broad significance especially if it is indeed the case that parties will be unable to democratically legitimize themselves solely through their governing function (the very feature that made them unique within the societal realm) as Peter prominently argued (Mair, 2009).

Looking at the wide range of Peter’s work more closely it becomes clear that he himself never abandoned the society-centered perspective on parties, irrespective of putting the state-centered perspective on parties on the map. We find evidence of this as early as in the Katz/Mair – Koole exchange of 1996 about the ‘cartel party model’ presented in *Party Politics* in 1995. In his 1996 critique of the latter, Koole (1996, pp. 513–514) stressed the parallels between parties, interest groups or churches in terms of growing state penetration on the one hand and the challenges of individualization on the other, suggesting the cartel party model – rather than capturing a genuine transformation of parties’ – might capture broader trends. This was read by Katz and Mair as confirmation of their argument they made for parties that could be generalized to a wider universe of organizations (1996, p. 528), also highlighting that their initial article had already presented party-state relations as not unlike relations between major interest groups and the state (Katz & Mair, 1995, p. 23). Indeed, comparisons across organizational types can also be found later, for instance, in his research on party membership decline highlighting fundamental challenges confronting parties, unions and churches alike (e.g. van Biezen, Mair, & Pogunkte, 2012).

## From civil society to the state and back – challenging the mainstream

Although looking at Peter’s work we find society-centered perspectives on parties alongside an increasingly prominent ‘statist perspective’, encompassing theoretical perspectives on ‘political organizations’ including parties, civic associations or interest groups as proposed by James Q. Wilson in his seminal book (1973) published half a century ago, that Peter held in high esteem, have not featured prominently in recent decades (but see Farrer, 2017; Fraussen & Halpin, 2018; Bolleyer 2024; Bolleyer & Correa, 2020). Yet that does not mean that research has not challenged the party- or state-centrism prevalent in party research since.

Starting with work challenging party-centrism, we find growing bodies of research on the evolving relations between parties and interest groups as well as between parties and social movements. Indeed, the study of party-interest group relations was directly inspired by cartel party theory as parties’ weakening ties to interest groups – especially unions – was one hypothesized symptom of organizational cartelization, i.e. one consequence of intensifying party-state relations alongside the decline of parties’ membership base (e.g. Allern & Bale, 2012; Allern et al., 2023). These roots in themselves already signal that while the study of party-interest group relations, by definition, does not keep parties separate from other organizations, it takes on board the assumptions about parties’ unique role towards the state, especially if the latter functions as a possible driver of party-group ties to become less institutionalized. Research on party-movement relations strongly shaped by sociology (for an early overview of this emerging research agenda, see Hutter, Kriesi, & Lorenzini, 2019) has not been influenced by cartel party theory in a similar way and not only, by definition, breaks with party centrism. It also challenges substantive claims towards a division of labor between movements as ‘real opposition’ and parties as ‘governors’ (Mair, 2013, pp. 88–89) by showing that some mainstream parties have strategically started to join forces with societal actors to exercise influence outside of those institutional areas to which they have privileged access (Borbáth & Hutter, 2021; Heinze & Weisskircher, 2022). Addressing such developments requires not only to bring in types of organizations other than parties but challenges dominant assumptions about mainstream parties becoming increasingly separated from the societal realm or relatedly an increasingly pronounced division of labor between parties (as part of the state apparatus) and societal organizations (Mair, 2013, pp. 84–85).

Work challenging substantive assumptions related to ‘state-centrism’ in party research stressing the centrality of parties’ relationship to the state is most clearly located within party research itself. Research on anti-establishment, challenger or niche parties deal with subsets of parties to which assumptions tailored to mainstream parties do not apply, not at least in earlier developmental phases (e.g. Meguid, 2008; De Vries & Hobolt, 2020). Broadening our notion of what defines political parties as a class of organization able to encompass mainstream and challengers alike, we return to Sartori (1976) and electoral participation as parties’ constitutive activity, irrespective of parties’ institutional access. These strands of research explicitly (re)diversify the dominant notion of political party used and challenge a ‘statist’ perspective on parties, in line with Peter’s later work dealing with the rise of populist challenger parties as one expression of citizens’ dissatisfaction with increasingly cartelized party systems. Bringing society back in, these parties represent a backlash against the mainstream’s turning into cartel parties, i.e. governors with little interest in and ability towards societal representation (Mair, 2002, 2013). That said, this research remains party-centric and maintains the boundaries between the study of parties and the study of other societal organizations. It therefore remains compatible with claims about parties’ distinctiveness, though this distinctiveness is conceptualized more inclusively, rooted in parties’ participation in electoral contests and not in the chance to place political personnel within state institutions.

This brief review has shown that by now different strands of research have challenged the ‘statist turn’ particularly prominent in the study of party organization. Most of these strands tend to challenge one of its two (often reinforcing) consequences, either accepting parties’ substantive uniqueness rooted in their special role in state institutions or accepting the need to separate them out analytically as a class of organization to be theorized and studied by itself. Research that explicitly tries to overcome both – by approaching parties from a societal perspective and by ceasing to study and theorize parties separate from other societal or political organizations – has remained rare (but see Farrer, 2017; Fraussen & Halpin, 2018; Bolleyer, 2024). The following section tries to illustrate how a more eclectic approach towards the study of parties embodied by Peter’s work as a whole – specifically a return to society-centered perspectives on parties – might be a worthwhile enterprise.

## From civil society to the state and back – parties as special but not unique

### Conceptualizing parties as part of a wider universe of organizations

For decades party research has been concerned with party decline and, relatedly, the decline of party democracy centering around arguments about parties’ decreasing ability to function as meaningful vehicles of representation, which especially Peter’s later work dealt with (Mair, 2009; 2013). Accounting for patterns of member activism (participation) and political engagement (representation) – two central organizational contributions to democracy – across a wider range of membership organizations will allow us to gain an alternative perspective on how contemporary parties as societal actors perform as compared to membership organizations facing the same societal challenges. Claims that such perspective might be fruitful are long-standing. Both party and group scholars have made comparisons between parties and other collective action organizations or, alternatively, between collective actors and more issue-specific and individualized channels for influence seeking in individualizing societies, wondering whether parties (or membership organizations more generally) might be an outdated form of organization.^3^ Systematic empirical evidence to examine such claims is still difficult to come by. To close this gap, we need to return to where Peter’s critique of party research started out from thirty years ago, a notion of parties as ‘voluntary associations which rely on at least a minimum of non-obligatory participation’ (Panebianco, 1988, p. 10; Wilson, 1973).

In the remainder of this essay, I build on a book-length study on the discrepancies between membership organizations’ democratic potential and their actual contributions to democracy (Bolleyer, 2024)^4^ to outline such society-centered perspective on parties, approaching them as one form of membership-based voluntary organizations alongside interest groups and service-oriented organizations. Parties are conceptualized as organizations with a voluntary (non-compulsory) membership that are private, separate (though not necessarily ‘autonomous’) from government, self-governing, non-profit distributing and have a formalized infrastructure.^5^ Doing so deliberately treats parties as part of ‘a wider universe’, in which they are a ‘special case of (…) many organizations heretofore regarded as primarily serving the function of linkage between society and the state’ (Katz & Mair, 1996, p. 528). Though encompassing membership organizations with a variety of primary missions, the organizations studied still share that they remain dependent on voluntary support of societal actors (Wilson, 1973), which is essential as it allows them – at least potentially – to function as venues for participation and vehicles of representation. Their basic set-up makes them directly comparable, whether they realize such potentials or not.

### Analyzing parties’ democratic contributions from a society-centered perspective: disaggregating the transmission belt

To conceptualize and analyse membership organizations’ democratic contributions from a society-centered perspective, it is useful to return to the classical notion of organizations as transmission belts. A membership organization corresponding to the latter is not only politically engaged but also held accountable by its members who have a say in central domains of decision-making. If (actively used) member control comes together with the organization’s external political engagement, we tend assume representation activity to be responsive to those societal interest that members care about. External political activity and intra-organizational participation – representation and participation – embodied by the ‘transmission belt’ correspond to two central functions that Dalton, Farrell, and McAllister (2011, p. 6) attributed to parties ‘as organizations’, notions we find in the group and non-profit literature as well (Knoke, 1990, p. 21; Jordan & Maloney, 2007, p. 2; van Deth & Maloney, 2012, p. 4). They therefore can be used as central normative yardsticks to assess membership-based CSOs’ democratic contributions generally.

Engagement in interest representation is defined as organization-level activity that involves the articulation of ‘politically relevant’ interests, i.e. interests that are created in response to perceived or anticipated effects of government action or inaction (Salisbury, 1984, pp. 64–65). Research has long stressed the complementary roles that parties, groups, and service organizations play in the aggregation and channeling of societal interests into the political and public sphere (Hutter et al., 2019, pp. 223–224; Wilson, 1973). Meanwhile, organizations composed of members by definition have the potential to have an active membership.^6^ One form of member activism materializes when groups are ‘substantially self-governing’, meaning intra-organizational practices emulate democratic procedures (Knoke, 1990, pp. 10–12). This particular form of member activism – *member control over decision-making* – is expected to enhance members’ capacity for self-governance and collective action, while fostering their political skills (Dekker, 2009, p. 228; Skocpol, 2013). Instead, members might participate in solidary activities offered by the organization or engage in organizational work such as supporting fundraising activities, participating in member recruitment or mobilizing support for petitions, whether organizational leaders are held accountable by members or not. Such *member involvement* (akin to volunteering in non-profit research) has been associated with a range of social benefits such as the cultivation of well-being and life-satisfaction through social interaction, the enhancement of human capital, the prevention of social atomization, trust, the mobilization and detection of unmet social needs and collective efforts to meet such needs (Hustinx, Cnaan, & Handy, 2010, pp. 417–418, 422). To assess membership organizations’ participatory benefits, both the granting of member control and the cultivation of member involvement need consideration.

Returning to the two overall yardsticks representation and participation, while engagement in interest representation behavior and the cultivation of active members (whether in terms of member control or involvement) can go together as suggested by the classical notion of transmission belt, they do not need to. Whether, when, and how parties, interest groups or civic associations are still able or ready to ‘perform’ as venues of participation and as vehicles for democratic representation simultaneously has become a wide-spread concern (van Deth & Maloney, 2010, p. 5; Bernhagen & Maloney, 2010, pp. 100–101; Kohler-Koch & Quittkat, 2013; Ahlquist & Levi, 2014; Bentancur, Rodríguez, & Rosenblatt, 2019). Particularly group scholars have stressed the rise of organizations granting no member control or even involvement that are politically active and very effectively so. Vice versa, we find organizations cultivating high levels of member control that are predominantly dedicated to social activities. This diversity suggests that to gain an understanding in membership organizations’ actual democratic contributions we need to disaggregate the ‘transmission belt’ and assess parties’ and groups’ participatory and representative contributions separately.

The former scenario – active interest representation without internal participation – is as applicable to parties as groups, and there are classical arguments in both literatures that suggest that external political activity might either not require or actually benefit from the absence of member participation, making the organization more unified and more forceful in pushing for its agenda (Schattschneider, 1942; Sartori, 1965; Schmitter & Streeck, 1999; Halpin, 2006). The reverse scenario – the absence of regular political engagement in face of member activism – seems less relevant to parties, because we tend to define parties by electoral participation, hence we presuppose some political activity to classify an organization as party to start with. This assumes that organizations that consider themselves as political parties regularly manage to nominate candidates for elections and do not fail already at the registration stage (Pedersen, 1982) and that their main mode of seeking political influence is running elections. While both assumptions apply well enough to major mainstream parties, casting the net more widely redirects our attention to a variety of other influence-seeking strategies parties as well as groups might employ crucial to establish linkages to audiences outside the institutional arena.^7^ In comparison to other membership organizations the question then poses itself whether the average party tends to specialize – in line with the vision of parties as ‘electoral vehicles’ – or, in the opposite, tends to engage in a wider range of these influence-strategies than the average group.

### A society-centered assessment of parties’ democratic contributions

When synthesizing existing party and group research, theoretical rationalizations are easy to come by why parties – more so than groups – might face incentives to cultivate member activism as well as a wider range of political activities. Starting with the participation yardstick, research suggests that the distinction between parties and groups feeds into different expectations of external audiences, which in different ways incentivize leaders to grant members control. Unlike groups (whether advocacy- or service-orientated), political parties usually recruit political candidates that participate in elections with the aim to win seats in parliament, to ultimately take over government and implement policies (Sartori, 1976). Consequently, parties play a more institutionally pre-defined role in democratic representation and as a consequence of this special status can be expected to face stronger normative pressures to replicate democratic standards within their organizations reinforced by citizens’ increasing ‘reluctance to choose among pre-packaged party-platforms’ than groups whose mode of engagement with constituencies can be much more varied (Kittilson & Scarrow, 2003, p. 59; Allern, 2010, pp. 93–94; Halpin, 2006, 2014).

Regarding leaders’ incentives to cultivate member involvement, the distinction between groups and parties can be expected to have similar implications. However, the rationale suggesting such difference follows a ‘material’ rather than ‘normative’ logic. While member involvement such as in organizational work is conducive to organizational self-maintenance irrespective of an organization’s primary purpose, to parties such involvement is more likely to function as a *critical* resource to assure central activities (e.g. Pfeffer & Salancik, 1978). Unlike groups, parties run elections whose regularity is institutionally prescribed. Furthermore, for parties an involved membership is particularly helpful as a pool of committed members willing to stand for office, a demanding form of involvement with – in most parties – offers little benefit, as in most cases there is no realistic chance of winning a seat. Interest groups and service-oriented organizations can be more selective regarding when and how they involve their membership (Holyoke, 2013; Hustinx, 2014).

Moving to political engagement, politicization, i.e. regular engagement in some political activity, such as electoral campaigning, is central to parties’ identity and thus less likely to be a ‘by product’ that can only be produced if selective incentives are provided as well, as Olson famously theorized for economic organizations (1965). This, however, does not mean that parties’ interest representation activities are necessarily focused on or restricted to electoral engagement. Given the broader set of roles attributed to parties in a democracy across various domains, both societal and institutional, they are not only more likely to be politicized than groups but also likely to engage in a broader range of political activities than other organizations to establish channels between citizens and the political sphere beyond the electoral arena.

To which extent do such expectations hold empirically? Table 1 summaries the findings of four analyses of survey data covering a total of 3265 membership organizations including parties, interest groups and service-orientated organizations^8^ across four European countries^9^ (Germany, Norway, UK, Switzerland) that formed part of a recent study analysing membership organizations’ democratic contributions in terms of member activism and political engagement (Bolleyer, 2024).^10^ Only explanatory variables are included in this summary of main findings that had significant effects in at least two of the four analyses. The four dependent variables – i.e. proxies for organizations’ varying contributions to participation and representation – were operationalized in the following fashion: To measure *Member Control* over decision-making I use an index that is based on three indicators from a survey question in which participants had to indicate how their organizations primarily make decisions in different areas. The index has been constructed by adding up (with equal weight) the following dimensions, which align closely with notions of intra-organization democracy: ‘Appointing board members or the executive’, ‘Appointing the chairperson or the leader’ and ‘Changing the statutory rules or the constitution’. Each component indicator has been coded as 1 when decisions in an organization were taken by consensus or by voting among members (indicating members’ direct control over decisions in the respective domains) and 0 when decisions were made otherwise (i.e. by the board, by the chairperson, by senior staff). Hence, the higher the index is, the more extensively members exercise direct control over central decisions. The measure for *Member Involvement* as the second form of member activism is based on a survey item (a five-point Likert scale) asking participants how involved their members are in organizational activities, 1 being not at all involved and 5 extremely involved.

**Table 1.** Patterns of member activism and political engagement in parties and groups.

	Normative Yardstick	Participation		Representation	
	*Empirical Dimensions*	*Member Activism*		*Political Activities*	
	Selected Explanatory Variables	*Member Control*	*Member Involvement*	*Politicization*	*Political Action Repertoire*
Primary purpose	**Party vs. Interest Group/ Service-oriented Organization**^11^		**+**	**+**	**+**
Features prominent in parties	Individual vs Corporate Membership Composition		−	−	−
	Outward Orientation vs. Member Interest Orientation		−	+	+
Central organizational characteristics	Membership Size	−	−		+
	Membership Instability			+	+
	Organizational Age	+			−
	Volunteer Staff		+		−
	Professionalization	−	+	+	+
	Bureaucratization		+	+	+

Notes: Reports only significant effects robust across different model specifications, − indicates negative relationship, + a positive relationship; cells are empty when no robust significant relationship (at least on 0.05 level) was found.

Both measures of organizations’ political engagement were based on the following survey item: ‘The table below lists a range of activities organizations can engage in to exercise political influence. Please indicate which activities your organization engages in nowadays.’ The 13 activities given as options deliberately covered a wide spectrum including political and partisan activities, conventional and unconventional forms of political participation, educational activities, activities directed towards government institutions as well as those targeting the public. For each activity organizations were asked to indicate whether they engage in it never, rarely, sometimes, often or very often. I considered a CSO as politically active – i.e. as *politicized* – if it engages in at least one or more of these 13 activities *either often* or *very often*. In contrast to *occasional* or *rare* engagement, this indicates the prioritization of political activity. If resources are regularly invested in the latter, this usually goes at the cost of other activities, i.e. forces CSOs to engage in trade-off decisions making choices between conflicting priorities central to the study’s overall theoretical framework. *Political Activity Repertoire* captures the intensity and diversity of such engagement based on the number of political activities an organization engages in often or very often, captured through an additive index ranging from 0 to 13.

Findings in Table 1 are presented with a focus on the party-group distinction measured by an item capturing whether organizations self-identified as party, interest group or service-orientated – in conjunction with features typical for parties (an outwards orientation towards the interests of wider societal constituencies rather than member interests; being composed of predominantly individual rather than corporate members). This enables us to evaluate whether parties’ democratic contributions as societal actors are likely to be more pronounced than those of membership organizations with different primary purposes.

As Table 1 indicates, being a party is, on average, associated with a more involved membership, while parties are, on average, also more likely to engage in political activities regularly and in a wider range thereof than both types of groups. Being a (self-declared) party has significant effects on both dimensions – member activism and political engagement – that are positive. Taking a society-centered perspective on parties thus provides evidence towards political parties’ special status among membership organizations both as participatory venues and vehicles for interest representation. The positive association with involvement is particularly important in a climate in which parties are generally unpopular and their importance as societal organizations is frequently questioned (e.g. Katz, 1990; Dalton & Weldon, 2005). Being a party is conducive to this form of member activism, even though being orientated towards the interests of wider societal interests – a feature typical for parties usually orientated towards voters – has a negative relationship with it. At the same time, being a party *per se* does not create incentives to be internally more democratic than being a group, an expectation that might have been too much inspired by research on mainstream parties that have been shown to embark on democratizing reforms (e.g. Gauja, 2017). Most minor parties, which form the majority of electorally active parties covered in the above analyses represent only very small sections of the electorate, have few or no seats and receive only little or no media attention. This, in turn, suggests very little public knowledge about their internal functioning, conditions that make it unlikely that parties grant more member control than they are ideologically or strategically inclined to. Similarly, linking this observation to cartel party theory, expectations towards parties to emulate democracy on the state level within their own organization might only arise once parties do become governors and occupy prominent positions within state structures, inviting those parties’ programmatic as well as structural assimilation (Katz & Mair, 2009, 2018).

Returning to the overall findings, whether members directly control internal decisions or not, member involvement as volunteering has benefits for the social fabric underpinning democratic regimes (Hustinx et al., 2010, pp. 417–418, 422). This suggests that despite organizational landscapes in contemporary democracies diversifying and offering citizens an ever-growing choice of venues to express their opinions and pursue their interests individually or collectively, political parties remain a central vehicle for member mobilization and engagement also in increasingly individualized democracies. This gains further weight when considering that involvement in parties – unlike involvement in service-oriented organizations – by definition supports the pursuit of political goals and thus contributes to interest representation.

That parties are, on average, more likely to be politicized and have a wider political action repertoire than service providers that are socially rather than politically orientated seems obvious. This is less the case when comparing parties to organizations such as interest groups also defined by a political mission (e.g. Wilson, 1973; Fraussen & Halpin, 2018). The literature on politicization has pointed out that – on the organization level – politicization can become manifest in an organization’s goals and its political action repertoire, two aspects that do not always go together (e.g. Zamponi & Bosi, 2018). The findings imply that having political goals more consistently translates into sustained political activity in the case of organizations that consider themselves as parties than those that consider themselves as interest groups. If so, (self-declared) parties are more likely to function as vehicles for political interest representation than (self-declared) interest groups.

This line of argument is substantiated given parties’ (on average) broader political action repertoires, suggesting that parties tend to provide more variegated channels into politics than other membership organizations. To give a concrete example, as compared to 49% of parties participating in the survey which use more than three political activities regularly, only 25.5% of interest groups and 19% of service-orientated organizations do so (the remaining organizations engage in fewer). Given the prominence of the notion of parties as ‘electoral vehicles’ in party research, this is no matter of course. Instead, the finding complements the well-established characterization of parties as programmatically more broad-ranging than issue-specific interest groups on the behavioral level, again underscoring the central role of parties in contemporary democracies. This is the case despite the above analyses’ sole focus on parties’ democratic contributions ‘as organizations’, leaving aside – from the start – their important contributions to democracy from ‘within government’ (Dalton et al., 2011, p. 6). Relatedly, parties tend to mobilize individual citizens whose voice is commonly considered more relevant and beneficial to the democratic process than those of corporate members (Jordan & Maloney, 2007, p. 195). That being predominantly composed of individual members has in itself negative associations with both member involvement and political engagement only underlines the relevance of their positive association with being a party, a type of organization predominantly dedicated to mobilizing individual citizens’ partisan interests.

## Conclusion

It is fair to say that Peter’s later work was pessimistic about the future of democracy and the role political parties might play in it (Mair, 2002, 2009, 2013). For sure, by changing our perspective away from major mainstream parties, we cannot challenge existing evidence about a growing disenchantment that citizens experience with these parties, particularly those that end up governing. But broadening our perspective to a wider range of parties and assessing them from a societal perspective in relation to alternative organizational forms of collective mobilization rather than some abstract or past ideal might help to qualify this pessimism somewhat. To do so it is essential to reflect upon how we define political parties as our ‘basic unit of thinking’ (Mair, 2008, p. 72). This might – from time to time – require us to step back and critically evaluate whether we have started to treat a conceptualization of party initially tailored to theorize and enlighten a specific dimension of how these organizations function and operate as a no longer questioned starting assumption.

The ‘statist turn’ in party research initially did exactly that. It expressed a critique of the traditional society-centered perspective on parties as private associations that had led to a systematic neglect of important dimensions of what major parties had become (Mair, 1994; Katz & Mair, 1995, 1996), thereby enriching the field of party studies with a range of new, still flourishing avenues for research (e.g. Bartolini & Daalder, 2014). Going back to the original text, Dick and Peter criticized tendencies in party research to ‘undervalue the extent to which differences between parties *may also be understood* by reference to their relations with the state’ (Katz & Mair, 1995, p. 6; italics added). A year earlier Peter had argued, more forcefully, that ‘the understanding of party organizational change and adaptation requires us to pay *as much attention, if not more*, to the linkage between party and the state as to the linkage between parties and civil society’ (Mair, 1994, p. 7, italics added).

Considering Peter’s eclectic and pluralist approach towards the study of politics generally (Bartolini & Daalder, 2014, p. 37), pushing the field in a new direction ultimately aimed at broadening our perspective on parties as our ‘basic unit of thinking’, not at replacing one dominant perspective by another. Various strands of research cutting across the boundaries of political science, sociology and organization research increasingly challenge different tendencies inherent in the state-centered approach to the study of parties that Peter’s work unintentionally invited and push the field back to more encompassing perspectives that his oeuvre in its entirety stands for (e.g. Farrer, 2017; Bolleyer, 2018; Fraussen & Halpin, 2018; Hutter et al., 2019; Allern et al., 2023). Pursuing this direction further by conceptualizing parties as comparable to other societal organizations – be those organizations interest groups, service-oriented organizations or protest movements – might paradoxically generate stronger evidence that parties can still be understood as ‘primus inter pares’ in the societal realm (Rosenblum, 2000a, 2000b), than insisting in their distinctive or even unique characteristics.

## Notes

1 Vice versa, a suitable definition needs to clearly distinguish parties from other politically active organizations. In many democracies, detailed state regulation is no distinct feature of parties anymore as increasingly complex regulatory regimes apply to other organizations in the civil society sector, be they advocacy- or service-orientated (Bolleyer, 2018).

2 Importance is easily indicated referring to the scale of electoral support, size of their membership or actual control over policy-making power in parliament and government.

3 See on this discussion, for instance, Lawson & Merkl, 1988; Daalder, 1992; Norris, 2002; Thomassen & van Ham, 2014; Ignazi, 2021.

4 The study is available open access here: https://fdslive.oup.com/www.oup.com/academic/pdf/openaccess/9780198884392.pdf. It is an output of the ERC-funded project Civilspace (https://www.cps-lmu.org/civilspace.html).

5 Members are individuals, organizations and institutions joining a CSO by accepting its mission and core values (Staggenborg, 1988, p. 586).

6 Member activism is defined by all activities through which members participate within CSOs, while passive membership is restricted to paying fees (Duverger, 1964: 90-116; Jordan & Maloney, 2007: 156).

7 Given ties to social movements are typical in this party family, the literature on Green parties has traditionally paid attention to this (Bolleyer, 2013).

8 The survey questionnaire was specifically designed to allow for the systematic comparison of different CSO types, i.e. partisan, advocacy and service-oriented.

9 Details on case/country selection, data collection and methods are provided in Chapter 3: https://fdslive.oup.com/www.oup.com/academic/pdf/openaccess/9780198884392.pdf.

10 The third normative yardstick that was integral to the framework in the book – the societal responsiveness of organizational behavior – is not included here. Being a party had no significant effects in the respective analyses (Bolleyer, 2024, p. 250).

11 Note that the reported effects hold when treating parties as reference group to interest groups and service-orientated organizations respectively.
